# Methoxyflavone derivatives modulate the effect of TRAIL-induced apoptosis in human leukemic cell lines

**DOI:** 10.1186/1756-8722-4-52

**Published:** 2011-12-21

**Authors:** Benjawan Wudtiwai, Bungorn Sripanidkulchai, Prachya Kongtawelert, Ratana Banjerdpongchai

**Affiliations:** 1 Department of Biochemistry, Faculty of Medicine, Chiang Mai University, Chiang Mai 50200, Thailand; 2 Center of Research and Development of Herbal Health Products, Faculty of Pharmaceutical Sciences, Khon Kaen University, Khon Kaen 40002, Thailand

**Keywords:** TRAIL, methoxyflavone derivatives, apoptosis, death receptor, mitochondrial pathway, human leukemic cells

## Abstract

**Background:**

Tumor necrosis factor-related apoptosis-inducing ligand (TRAIL) induces apoptosis in various tumor cells, but does not affect normal cells or human leukemic cells, such as MOLT-4 and U937 cells, which are relatively resistant to TRAIL. Three flavonoids extracted from the rhizome of *K. parviflora *were 5,7-dimethoxyflavone (DMF), 5,7,4'-trimethoxyflavone (TMF) and 3,5,7,3',4'-pentamethoxyflavone (PMF), and synthetic flavonoids including 5-methoxyflavone (5-MF) and 2'-methoxyflavone (2"-MF) were chosen for testing in this study. The aims of this study were to examine whether the treatment of TRAIL-resistant leukemia MOLT-4 and U937 cells, with methoxyflavone derivatives could enhance the apoptotic response and to identify the mechanism involved.

**Methods:**

The cytotoxic effect of methoxyflavone (MF) derivatives in MOLT-4, U937 and peripheral blood mononuclear cells (PBMCs) was analyzed by the MTT assay. The induction of apoptosis and the reduction of mitochondrial transmembrane potential (ΔΨm) after staining with annexin V FITC and propidium iodide (PI), and 3,3'-dihexyloxacarbocyanine iodide (DiOC_6_), respectively, were performed using flow cytometry. ROS production was determined by staining with 2',7'-dichlorofluorescin diacetate and processed with a flow cytometer. DR4, DR5, cFLIP, Mcl-1, BAX and Bid expression were demonstrated by immunoblotting. Caspase-8 and -3 activities were determined by using IETD-AFC and DEVD-AFC substrates and the fluorescence intensity was measured.

**Results:**

All methoxyflavone derivatives were cytotoxic to MOLT-4, U937 cells and PBMCs, except DMF, TMF and PMF were not toxic to PBMCs. All MF derivatives induced human leukemic MOLT-4 cell apoptosis, but not in U937 cells. Percentage of MOLT-4 cells with (ΔΨm) was increased when treated with DMF, TMF, PMF, 5-MF and 2'-MF in the presence of TRAIL. 5-MF and 2'-MF enhanced TRAIL-induced apoptosis through the up-regulation of both DRs and the down-regulation of cFLIP and Mcl-1. Bid was cleaved and BAX was up-regulated, followed by the activation of caspase-8 and -3. Oxidative stress was also increased. 2'-MF gave the same result compared with 5-MF but with a less effect.

**Conclusion:**

Methoxyflavone derivatives enhanced TRAIL-induced apoptosis in human leukemic MOLT-4 cells through the death receptors and mitochondrial pathways.

## Introduction

Tumor necrosis factor (TNF)-related apoptosis-inducing ligand receptors are type II transmembrane proteins. They belong to TNF-R superfamily, having a short cytoplasmic N-terminal domain and a long C-terminal extracellular receptor. They include TRAIL-R1 (DR4) and TRAIL-R2 (DR5), which bind to ligands and induce apoptosis. TRAIL-R3 (decoy receptor 1) and TRAIL-R4. (decoy receptor 2), however, are non-apoptosis-inducing receptors, because they lack a functional cytoplasmic death domain [1]. TRAIL selectively induces apoptosis in a variety of tumor cells, but is relatively non-toxic to normal cells. Because of this, it is currently being used in clinical trials for cancer treatment in combination with various chemotherapeutic agents [2]. However, some tumor cells have been shown to be resistant to TRAIL, such as MOLT-4 and U937 cells [3].

Methoxyflavones (MF) have been reported to contain more chemopreventive activity than flavones [4]. Methoxyflavone (MF) derivatives are groups of flavonoids containing various numbers of methoxy moieties, such as 2'-methoxyflavone (2'-MF), 5-methoxyflavone (5-MF), 5,7-dimethoxyflavone (DMF), 5,7,4'-trimethoxyflavone (TMF), and 3,5,7,3',4',-pentamethoxyflavone (PMF). Reported plant sources of these flavanoids include TMF, 5,7,3',4'-tetraMF, 3,5,7,4'-tetraMF from *Kaempferia parviflora *[5,6]; and 5,3'-dihydroxy-3,6,7,8,4'-pentamethoxyflavone (DH-PMF) from *Gardenia obtusifolia *[7]. The bioactivities of MF derivatives include anti-inflammatory (5,7-DMF), anti-malarial (TMF and 5,7,3',4'-tetraMF), antifungal (3,5,7,4'-tetraMF) [5]; antagonistic to aryl hydrocarbon receptor (6,2',4'-TMF) [8] and apoptosis inducing properties (5,3'-dihydroxy-3,6,7,8,4'-pentamethoxyflavone) [7].

Flavonoids can induce apoptosis when combined with TRAIL [9]. Thus, the aims of this study were to compare the cytotoxic effects of methoxyflavone derivatives on apoptotic induction alone and combined with TRAIL in MOLT-4 and U937 cells, and to elucidate the mechanisms of cell death.

## Materials and methods

### Chemicals and reagents

5,7-Dimethoxyflavone (DMF), 5,7,4'-trimethoxyflavone (TMF) and 3,5,7,3',4'-pentamethoxyflavone (PMF), which were isolated and purified from rhizomes of *K. parviflora *as previously described [6]. 5-Methoxyflavone, 2'-methoxyflavone, histopaque, MTT (3-(4,5-dimethyl)-2,5-diphenyl tetrazolium bromide, propidium iodide (PI), 3,3'-dihexyloxacarbocyanine iodide (DiOC_6_) and 2',7'-dichlorofluorescin diacetate (DCFH-DA) were obtained from Sigma-Aldrich (St. Louis, MO, USA). TRAIL was obtained from R&D system, USA. RPMI-1640 medium, DEVD-AFC (Asp-Glu-Val-Asp-7-amino-4-trifluoromethylcoumarin) and IETD-AFC (Ile-Glu-Thr-Asp-amino-4-trifluoromethylcoumarin) were obtained from Invitrogen, USA. Mouse monoclonal antibodies to Mcl-1, BAX and rabbit polyclonal antibody to Bid, cFLIP and horseradish peroxidase (HRP) conjugated secondary antibodies were purchased from Abcam, Cambridge, UK. Mouse monoclonal antibodies to beta-actin, DR4 and DR5 were obtained from Santa Cruz Biotechnology, USA. SuperSignal West Pico Chemiluminecent Substrate was obtained from Pierce, Rockford, IL, USA. Annexin V-Fluos staining kit and complete mini protease inhibitor cocktail was obtained from Roche, Basel, Switzerland.

### Cell culture

Human lymphoblastic leukemic MOLT-4 and monocytic U937 cells were gifts from Professor Watchara Kasinroek (Faculty of Associated Medical Sciences, Chiang Mai University). Peripheral blood mononuclear cells (PBMCs) were donated from healthy volunteers. PBMCs were isolated from heparinized blood by density gradient centrifugation using histopaque according to standard protocols. The blood was obtained from adult volunteers with Institutional Review Board approval, at Faculty of Medicine, Chiang Mai University. The cells were cultured in RPMI-1640 medium with 25 mM NaHCO_3_, 20 mM HEPES, 100 units/mL penicillin, 100 μ g/mL streptomycin and supplemented with 10% fetal bovine serum. The cell lines were grown at 37°C in a 5% CO_2 _atmosphere. The PBMCs and human leukemic cells (1 × 10^6^) were treated with MF derivatives at indicated concentrations and durations. MF derivatives were dissolved in DMSO as a vehicle and the maximal volume used did not exceed 10 μ l/ml of media.

### Cytotoxicity test

Following MF derivative treatment, cell viability was assessed by the MTT (3-(4,5-dimethyl)-2,5-diphenyl tetrazolium bromide) assay [10]. This method is based on the ability of viable cells to reduce MTT and form a blue formazan product. MTT solution (sterile stock solution of 5 mg/ml) was added to cell suspension at a final concentration of 100 μ g/ml and the solution incubated for 4 h at 37°C in a humidified 5% CO_2 _atmosphere. The medium was then removed and cells were treated with DMSO for 30 min. The optical density of the cell lysate was measured at 540 nm, with a reference wavelength of 630 nm, using a microtiter plate reader (Biotek, USA). The number of viable cells was calculated from the number of untreated cells, and the data were expressed as percent cell viability.

### Apoptosis assay

After treatment with methoxyflavone derivatives at a concentration of IC_20 _for 0, 3, 6, 12, 18 and 24 h, the cells were washed with PBS and centrifuged at 200 × g for 5 minutes and suspended in 100 μ l of binding buffer from a kit containing annexin V-FITC and PI, for 15 min. The samples were analyzed using a flow cytometer (Beckton Dickinson, USA).

### Determination of mitochondrial transmembrane potential and ROS production

To measure mitochondrial membrane potential and intracellular ROS, either 40 nM 3,3'-dihexyloxacarbocyanine iodide (for mitochondrial transmembrane potential determination) or 5 μM 2',7'-dichlorofluorescin diacetate (for ROS detection) were added for 15 min at 37°C and the cells were then subjected to flow cytometry.

### Assay of caspase-3 and caspase-8 activity

Cleavage of the fluorogenic peptide substrates DEVD-AFC and IETD-AFC was used to assay caspase-3-like and caspase-8-like enzyme activity. Cell lysates (1×10^6 ^cells) and substrate (50 μM) were combined in a standard reaction buffer and added to a 96-well plate. Enzyme-catalyzed release of AFC was measured by a fluorescence plate reader (Bio-tek, USA) using 355 nm excitation and 460 nm emission wavelengths.

### Western blot analysis

To obtain a cytosolic-rich fraction, MF derivative-treated cells were harvested and washed once in ice cold PBS and incubated at 4°C for 10 min with ice-cold cell lysis buffer (137 mM NaCl, 15 mM EGTA, 0.1 mM Na_2_VO_4_, 15 mM MgCl_2_, 1% Triton X-100, with complete mini protease inhibitor cocktail). The cell suspension was centrifuged at 20,000 × *g *for 20 min. The supernatant was collected as the cytosolic-rich fraction. Protein concentration of the cytosolic-rich fraction was determined by the Bradford method. Cytosolic proteins (50 μg) were separated by 17% SDS-PAGE and transferred onto nitrocellulose membranes. After treating with 5% non-fat milk in TBS containing 0.2% Tween-20 (blocking buffer), membranes were incubated with mouse monoclonal antibodies to DR4, DR5, BAX and Mcl-1 and rabbit polyclonal antibody to Bid and cFLIP. For detection, appropriate horseradish peroxidase (HRP) conjugated secondary antibodies were used at 1:20,000 dilution. Protein bands were visualized on X-ray film with SuperSignal West Pico Chemiluminecent Substrate.

### Statistical analysis

Results were expressed as mean ± S.D. (standard deviation). Statistical difference between control and treated group was determined by a nonparametric one-way ANOVA (Kruskal Wallis test) with a limit of *p *< 0.05 in three independent experiments. For comparison between two groups, data were analyzed using Student's *t*-test.

## Results

### Cell cytotoxicity and apoptosis induction

**Figure 1:**
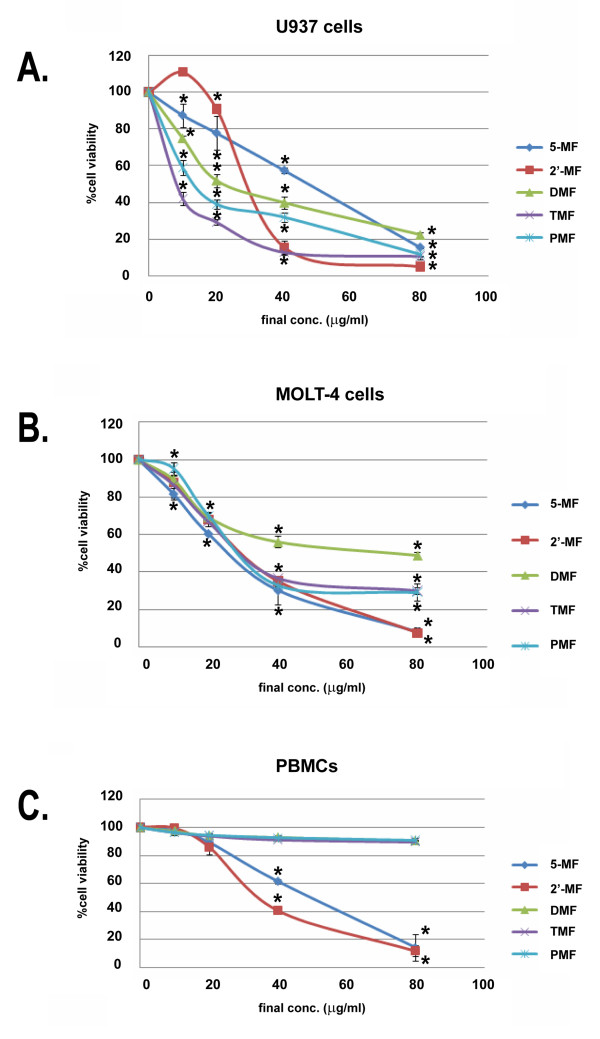
**Cytotoxic effects of DMF, TMF, PMF, 5-MF and 2'-MF on human leukemic MOLT-4, U937 cell lines and PBMCs**. MOLT-4 or U937 or PBMC cells were treated with DMF or TMF or PMF, 5-MF or 2'-MF at various concentrations for 24 h, then the cell viability was determined by the MTT assay. *, *p *< 0.05, compared with control.

**Figure 2:**
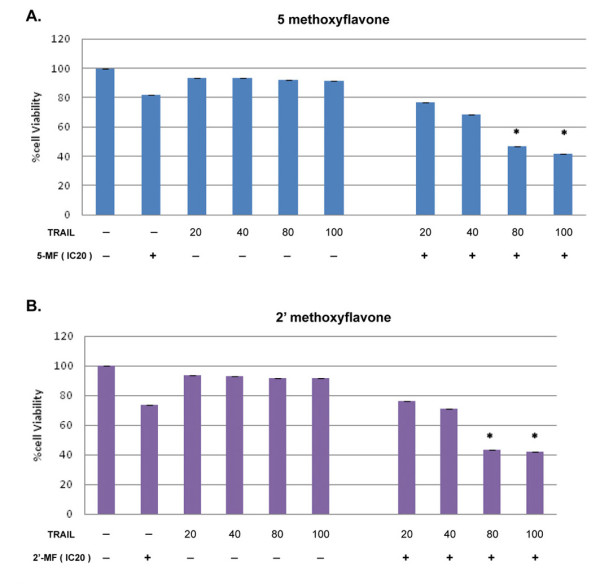
**Synergistic effects of 5-MF and 2'-MF when combined with TRAIL on MOLT-4 cytotoxicity**. The cells were treated with IC_20 _of 5-MF (A) or 2'-MF (B) for 24 h and then with various concentrations of TRAIL for 24 h, then cell viability was determined by the MTT assay and compared with MF alone or TRAIL alone. *, *p *< 0.05, compared with control.

All five MF derivatives were toxic to MOLT-4 and U937 cells with IC_50 _values as shown in Figure 1 and Table 1. DMF, TMF and PMF were not cytotoxic to PBMCs whereas 5-MF and 2'-MF were also toxic to PBMCs (Figure 1). TRAIL was not cytotoxic to U937 and MOLT-4 cells, viz. both cell lines were resistant to TRAIL (Figure 2). The IC_20 _level of MF derivatives was chosen for further experiments. In TRAIL combination with MF, MOLT-4 cells were induced to die more in a dose response manner (Figure 2).

**Table 1:** IC_50 _values of five methoxyflavone derivatives (2'-methoxyflavone, 5-methoxyflavone, DMF, TMF and PMF) in U937 and MOLT-4 cells

Methoxyflavone derivatives	IC_50 _values (μ g/ml)*	
	
	U937 cells	MOLT-4 cells
2'-MF	31.61 ± 0.68	30.11 ± 0.16

5-MF	46.70 ± 1.60	26.39 ± 2.22

DMF	24.56 ± 2.80	48.13 ± 5.12

TMF	12.95 ± 0.48	30.44 ± 0.52

PMF	19.28 ± 0.55	31.16 ± 2.22

*Means and standard deviations are calculated from triplicates of three independent experiments.

**Figure 3:**
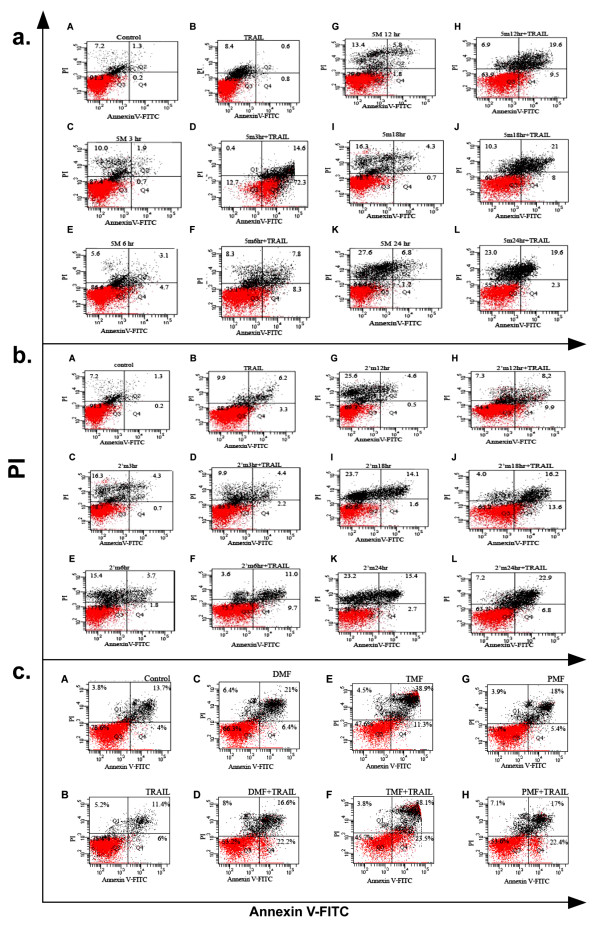
**Dot plot analysis of 5-MF or 2'-MF or DMF or TMF or PMF-treated MOLT-4 cells in the presence or absence of TRAIL**. The concentrations of 5-MF and TRAIL used were at IC_20 _levels. (a) The cells were untreated (A), treated with TRAIL for 24 h (B), 5-MF for 3 h (C), 5 MF for 3 h and TRAIL for 24 h (D), 5-MF for 6 h (E), 5-MF for 6 h and TRAIL for 24 h (F), 5-MF for 12 h (G), 5-MF for 12 h and TRAIL for 24 h (H), 5-MF for 18 h (I), 5-MF for 18 h and TRAIL for 24 h (J), 5-MF for 24 h (K) and 5-MF for 24 h and TRAIL for 24 h (L). (b) The MOLT-4 cells were untreated (A), treated with TRAIL for 24 h (B), 2'-MF for 3 h (C), 2'-MF for 3 h and TRAIL for 24 h (D), 2'-MF for 6 h (E), 2'-MF for 6 h and TRAIL for 24 h (F), 2'-MF for 12 h (G), 2'-MF for 12 h and TRIAL for 24 h (H), 2'-MF for 18 h (I), 2'-MF for 18 h and TRIAL for 24 h (J), 2'-MF for 24 h (K) 2'-MF for 24 h and TRAIL for 24 h (L). (c) The MOLT-4 cells were untreated (A), treated with TRAIL for 24 h (B), DMF for 24 h (C), DMF for 24 h plus TRAIL for 24 h (D), TMF for 24 h (E), TMF for 24 h plus TRAIL for 24 h (F), PMF for 24 h (G) and PMF for 24 h plus TRAIL for 24 h (H). The cells were stained with annexin V-FITC and PI, and processed by using flow cytometer. The percentages of early, late apoptotic cells and necrotic cells are shown in the right lower quadrant, right upper quadrant and left upper quadrant, respectively.

5-MF induced MOLT-4 cell apoptosis mostly at 3 h when combined with TRAIL for 24 h (Figure 3a) and 2'-Methoxyflavone induced apoptosis (the time dependence is shown in Figure 3b). Percent apoptotic cells increased when combined with TRAIL compared to the absence of TRAIL. Meanwhile DMF, TMF and PMF also induced MOLT-4 cell apoptosis synergistically to TRAIL (Figure 3c). Notably, all five MF derivatives and TRAIL synergistically induced MOLT-4 cell apoptosis, but this phenomenon did not occur in U937 cell line (data not shown).

### Reduction of mitochondrial transmembrane potential (MTP)

**Figure 4:**
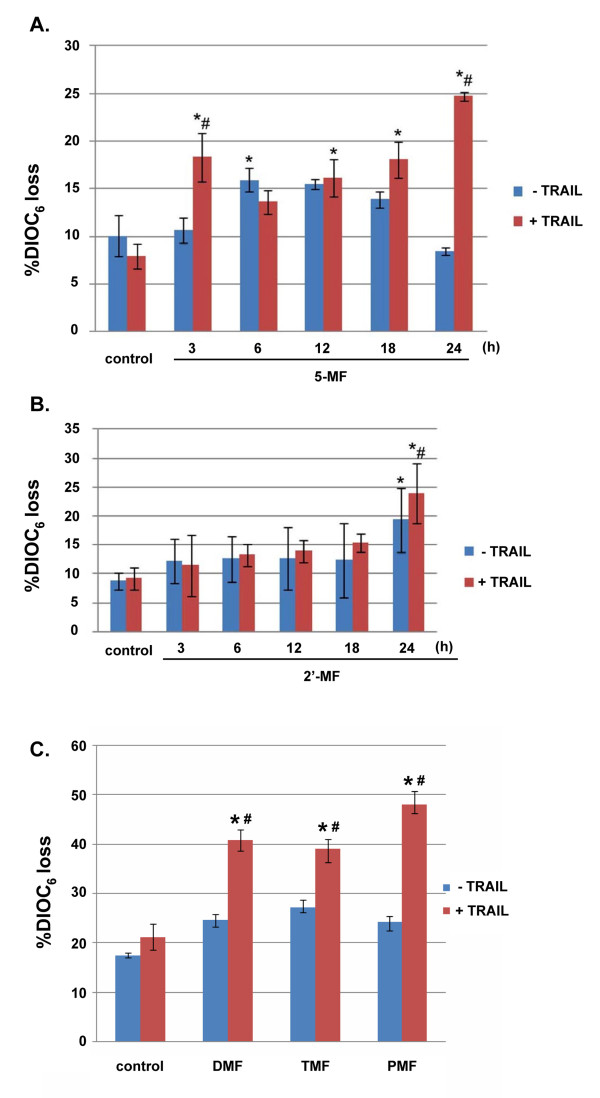
**Reduction of mitochondrial transmembrane potential in MOLT-4 cells treated with MF derivatives in the presence or absence of TRAIL**. The cells were treated with 5-MF at IC_20 _level (A) or 2'-MF at IC_20 _level (B) or DMF at IC_20 _level, TMF at IC_20 _level, PMF at IC_20 _level (C) for various times of incubation and then with or without TRAIL (IC_20_) for 24 h. After treatment the cells were incubated with DiOC_6 _followed by flow cytometry as described in the Methods. *, *p *< 0.05, compared with control**, #**, *p *< 0.05 compared to TRAIL alone.

The percentage of MOLT-4 cells (treated with 5-MF and/or TRAIL) with reduction of MTP was increased more than those treated with 5-MF alone at 3, 6, 12, 18 and 24 h (Figure 4A). The combined treatment also increased the cells with mitochondrial transmembrane reduction compared to TRAIL alone.

For the 2'-MF treatment for 24 h without TRAIL, percent cells with reduced mitochondrial transmembrane potential was increased compared to other incubation time points. The percentage of cells with loss of mitochondrial transmembrane potential significantly increased when treated with TRAIL for 24 h compared to TRAIL alone (control) as shown in Figure 4B.

For DMF, TMF and PMF treatment for 24 h in combination with TRAIL for another 24 h, the percentage of cells with reduction of mitochondrial transmembrane potential significantly increased when compared with control or TRAIL alone (Figure 4C).

### ROS production

**Figure 5:**
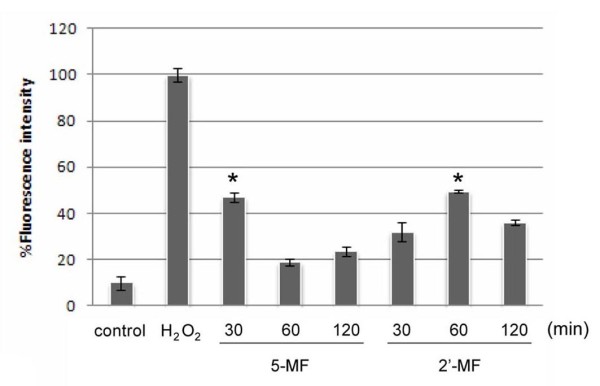
**Effect of 5-MF and 2'-MF on ROS production in MOLT-4 cells**. Cells were treated with 5-MF or 2'-MF for 30, 60 and 120 min and incubated with DCFH_2_-DA. The fluorescence intensity was measured by a flow cytometer. Positive control is H_2_O_2 _treatment at 3%H_2_O_2 _for 3 min. *, *p *< 0.05, compared with control.

Curcumin [11] and zerumbone [12] could induce cancer cells to undergo apoptosis via the excessive production of ROS. This led to the investigation of the ROS production in MF derivative (5-MF and 2'-MF) treatment in the MOLT-4 cells. When MOLT-4 cells were treated with 5-MF and measured for ROS production, the fluorescence intensity of DCF increased, with the maximum effect at 30 min treatment. For incubation with 2'-MF, ROS was greatest at 60 min treatment (Figure 5).

### The expression of TRAIL receptors (DR4 and DR5) and apoptosis-related proteins

**Figure 6:**
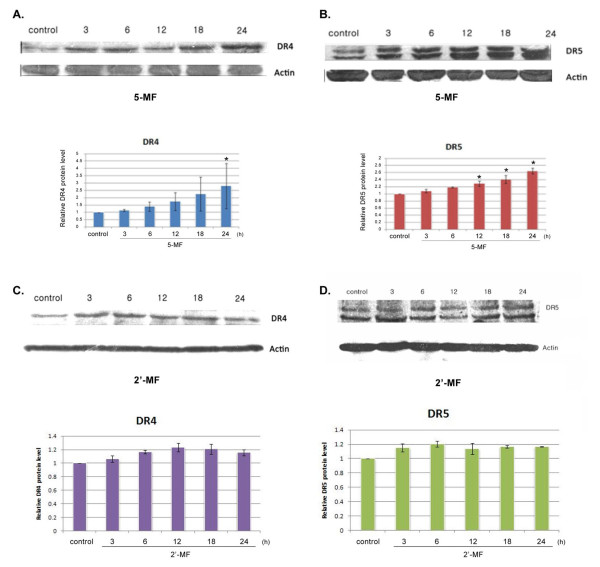
**Effect of 5-MF and 2'-MF on DR4 and DR5 expression in MOLT-4 cells**. The cells were treated with each MF derivative (at IC_20 _level) for various times and then treated with TRAIL (IC_20_) for 24 h. The whole-cell extracts were prepared and analyzed by Western blotting using anti-DR4 and anti-DR5 antibodies. DR4 (A) and DR5 (B) expression of MOLT-4 cells treated with 5-MF are shown. The cells were also treated with 2'-MF for various times of incubation and TRAIL for 24 h, DR4 (C) and DR5 (D) expression are shown. The intensity of bands was quantified by densitometry. *, *p *< 0.05, compared with control.

To determine how 5-MF and 2'-MF could facilitate TRAIL-induced apoptosis, the effects of both MF derivatives on TRAIL receptor (DR4 and DR5) expression were determined. MOLT-4 cells were treated with 5-MF or 2'-MF for various incubation times. The whole cell extracts were prepared and examined for the expression of DR4 and DR5. 5-MF induced both DR4 and DR5 (Figure 6A and 6B) expression in a time dependent manner, whereas the expression of DR4 and DR5 induced by 2'-MF was unaltered (Figure 6C and 6D). These results indicated that 5-MF up-regulated the expression of both DR4 and DR5.

**Figure 7:**
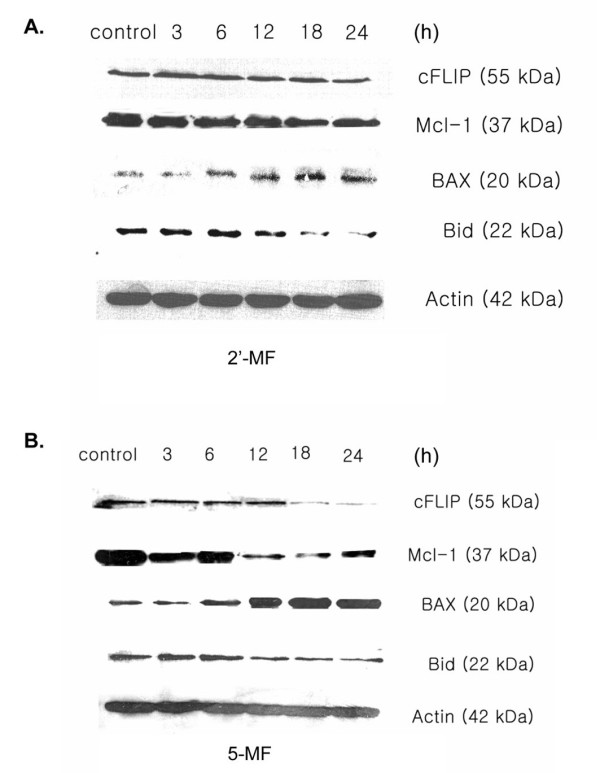
**Effect of 2'-MF and 5-MF on apoptosis-related protein expression in MOLT-4 cells**. Immunoblot analysis was performed to determine the expression levels of apoptosis-related proteins (c-FLIP, Mcl-1, BAX and Bid) after treatment with 2'-MF (A) and 5-MF (B) for various times. The immunoblots are representatives of 3 independent experiments with similar results.

The treatment with 5-MF at various times (3, 6, 12, 18 and 24 h) resulted in a time dependent reduction in the levels of antiapoptotic proteins cFLIP, Mcl-1 and an increase in the proapoptotic protein BAX (Figure 7). BH3 domain-only protein Bid (proform, 22 kDa) was reduced due to the cleavage to obtain the truncated Bid (tBid, 15 kDa). The 2'-MF treated cells had a lesser effect on the reduction of Bid, antiapoptotic protein cFLIP and Mcl-1 and an increase of BAX compared to 5-MF (Figure 7).

### Caspase-8 and -3 activation

**Figure 8:**
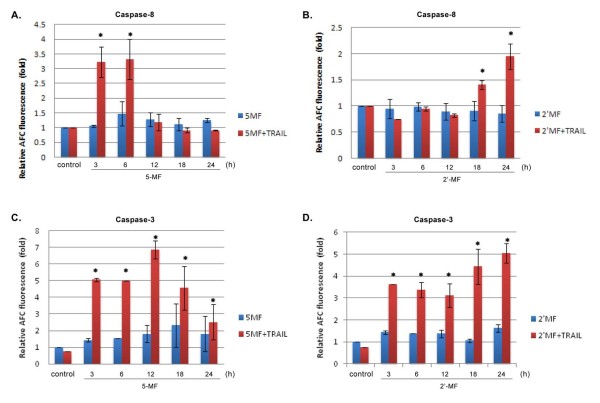
**Effect of 5-MF and 2'-MF on caspase-8 and -3 activities in MOLT-4 cells**. Caspase-8 activity was measured in 5-MF- (A) and 2'-MF- (B) treated cells for various times in the presence or absence of TRAIL for 24 h. The IETD-AFC substrate was cleaved and produced fluorescence. Caspase-3 activity was measured in 5-MF- (C) and 2'-MF- (D) treated cells for various times and with or without TRAIL for 24 h. The DEVD-AFC substrate was used. *N*-fold increase of fluorescence intensity was compared to that of untreated (control) cells, which was calculated to be 1. *, *p *< 0.05, compared with control.

Caspase-8 is activated by association with death ligand and receptor activation via the extrinsic pathway. In MOLT-4 cells treated with 5-MF and TRAIL, caspase-8 activity increased at 3 and 6 h of 5-MF treatment (Figure 8A). For the 2'-MF and TRAIL treatment, the caspase-8 activity increased between 18 and 24 h (Figure 8B). When MOLT-4 cells were treated with MF derivatives alone, caspase-8 activity did not change. In the presence of TRAIL, 5-MF and 2'-MF induced MOLT-4 cell apoptosis via the extrinsic pathway by activation of caspase-8 activity.

Caspase-3 plays a central role in the apoptotic cascade. When MOLT-4 cells were treated with 5-MF combined with TRAIL, caspase-3 activity increased at 3, 6, 12, 18 and 24 h of treatment (Figure 8C). MOLT-4 cells treated with 2'-MF and TRAIL, had increased caspase-3 activity at 3, 6, 12, 18 and 24 h of treatment (Figure 8D). However, when the cells were treated with MF derivatives alone, caspase-3 activity was unaltered. In the combined treatment, MOLT-4 cells were activated to undergo apoptosis via caspase-3 activation.

## Discussion

Recombinant human TRAIL has been recently recommended for clinical trials in the treatment of human cancer [13]. It selectively kills cancer cells while leaving normal cells unharmed [14]. However, some cancer cells are resistant to the TRAIL-induced apoptosis, such as human leukemic U937 and MOLT-4 cells [3,15]. We found that the methoxyflavone derivatives, DMF, TMF, PMF, 5-MF and 2'-MF could facilitate TRAIL-induced apoptosis in MOLT-4 cells (Figures 3 and 4). The apoptotic cell death was confirmed by the externalization of phosphatidylserine to the outer membrane of apoptotic cells [16]. Ionizing radiation also sensitizes human leukemic MOLT-4 cells to TRAIL-induced apoptosis [17].

5-MF and 2'-MF are methoxyflavones that are commercially available where as DMF, TMF and PMF were purified from rhizomes of *K. parviflora*. All five MFs were able to induce and enhance the apoptosis induced by TRAIL via the mitochondrial pathway (Figure 4). The BAX proteins, which form homodimers on the mitochondrial membrane, increased in expression, indicating mitochondrial pathway involvement. ROS production also occurred in the MF-induced apoptosis, suggesting that it might involve the mitochondria (Figure 5). Fluorescence intensity was high at 30 min for 5-MF and at 60 min for 2'-MF then it decreased afterwards. The reason for this phenomenon might be that each methoxyflavone derivatives could stimulate the ROS production with the peaks at different rates. However, the mechanism remains to be clarified.

Mcl-1 is a BH-multidomain member of the Bcl-2 family that exhibits potent antiapoptotic activity and plays a particularly important role in the survival of malignant hematopoietic cells [18]. Mcl-1 modulates apoptosis through multiple mechanisms, including interactions with proapoptotic members of the Bcl-2 family such as BH3-only domain proteins, for example, tBid [19]. Cooperation between activation of the intrinsic and extrinsic apoptotic pathways has been extensively described [20]. Evidence that Mcl-1 plays a role in controlling apoptosis by binding active Bid (tBid) therefore provides a theoretical basis for the observed synergism between MF derivatives and TRAIL. For example, in receptor-mediated induction of apoptosis, activation of Bid represents a critical component of the cascade. Following activation of procaspase-8 at the level of the death inducing signaling complex (DISC), death signals are transmitted to the mitochondria via cleavage of Bid to generate tBid. tBid interacts with BAX and Bak to promote their oligomerization and insertion into the outer mitochondrial membrane, leading to mitochondrial outer membrane permeabilization.

5-MF induced both DR4 (Figure 6A) and DR5 (Figure 6B) in a time-dependent manner but no change was observed for 2'-MF. To confirm this, we observed that 5-MF induced both DR4 and DR5 expression on the cell membrane using immunocytochemistry (data not shown).

In 5-MF/TRAIL- and 2'-MF/TRAIL-treated cells, caspase-8 was activated. At this level, the most potent inhibitor of caspase-8 is cFLIP, which is recruited along with procaspase-8 and FADD/TRADD to the DISC. cFLIP is a short-lived protein structurally related to procaspase-8 but lacking enzymatic activity [21]. 5-MF and 2'-MF treatment induced a dramatic decrease in cFLIP, which facilitated the activation of the extrinsic cascade. It is possible that the simultaneous down-regulation of Mcl-1 and cFLIP by 5-MF and 2'-MF might provide a mechanism by which TRAIL-resistant leukemia MOLT-4 cells were sensitized to these MF-derivatives. However, the signaling effect of 2'-MF was less than that of 5-MF.

Clinical application of DMF, TMF, PMF, 5-MF and 2'-MF is possible, although both 5-MF and 2'-MF were toxic to PBMCs. The IC_20 _concentration levels of 5-MF and 2'-MF induced human leukemic cell apoptosis with no toxicity to normal cells. However, even though these two methoxyflavone derivatives had a high potential capacity to kill cancer cells, especially in the TRAIL resistant human leukemic MOLT-4 cell type, the investigation in an *in vivo *model is needed before clinical trials.

In conclusion, 5-MF could enhance TRAIL-induced apoptosis through the up-regulation of both DRs and the down-regulation of cFLIP and Mcl-1, followed by the cleavage of Bid and activation of BAX. The caspase-8 was activated through the extrinsic pathway, and followed by activation of caspase-3. BAX oligomerization at the mitochondrial membrane led to the reduction of mitochondrial transmembrane potential. The mitochondrial pathway was also involved in activating caspase-3. ROS production might be the result of mitochondrial injury or the cause of mitochondrial signaling. 2'-MF had a similar but lesser effect on the apoptotic signaling pathway compared to 5-MF. DMF, TMF and PMF could also synergistically enhance TRAIL-induced apoptosis via mitochondrial pathway.

## Competing interests

The authors declare that they have no competing interests.

## Authors' contributions

RB, BW and PK conceived, designed and implemented the study, and RB drafted the manuscript. BS isolated and purified DMF, TMF and PMF.

## References

[B1] Ashkenazi A Dixit VM Apoptosis control by death and decoy receptors Curr Opin Cell Biol 1999 11 255 260 10.1016/S0955-0674(99)80034-9 10209153

[B2] Wang S El-Deiry WS TRAIL and apoptosis induction by TNF-family death receptors Oncogene 2003 22 8628 8633 10.1038/sj.onc.1207232 14634624

[B3] Lee NS Cheong HJ Kim SJ Kim SE Kim CK Lee KT Park SK Baick SH Hong DS Park HS Won JH Ex vivo purging of leukemia cells using tumor-necrosis-factor-related apoptosis-inducing ligand in hematopoietic stem cell transplantation Leukemia 2003 17 1375 1383 10.1038/sj.leu.2402960 12835727

[B4] Walle T Ta N Kawamori T Wen X Tsuji PA Walle UK Cancer chemopreventive properties of orally bioavailable flavonoids--methylated versus unmethylated flavones Biochem Pharmacol 2007 73 1288 1296 10.1016/j.bcp.2006.12.028 17250812 PMC1868573

[B5] Yenjai C Prasanphen K Daodee S Wongpanich V Kittakoop P Bioactive flavonoids from *Kaempferia parviflora* Fitoterapia 2004 75 89 92 10.1016/j.fitote.2003.08.017 14693228

[B6] Sutthanut K Sripanidkulchai B Yenjai C Jay M Simultaneous identification and quantitation of 11 flavonoid constituents in *Kaempferia parviflora *by gas chromatography J Chromatogr A 2007 1143 227 233 10.1016/j.chroma.2007.01.033 17266972

[B7] Murray IA Flaveny CA DiNatale BC Chairo CR Schroeder JC Kusnadi A Perdew GH Antagonism of aryl hydrocarbon receptor signaling by 6,2',4'-trimethoxyflavone J Pharmacol Exp Ther 2010 332 135 144 10.1124/jpet.109.158261 19828881 PMC2802483

[B8] Phromnoi K Reuter S Sung B Limtrakul P Aggarwal BB A Dihydroxy-pentamethoxyflavone from Gardenia obtusifolia suppresses proliferation and promotes apoptosis of tumor cells through modulation of multiple cell signaling pathways Anticancer Res 2010 30 3599 3610 20944143 PMC3142747

[B9] Hasegawa H Yamada Y Komiyama K Hayashi M Ishibashi M Yoshida T Sakai T Koyano T Kam TS Murata K Sugahara K Tsuruda K Akamatsu N Tsukasaki K Masuda M Takasu N Kamihira S Dihydroflavonol BB-1, an extract of natural plant *Blumea balsamifera*, abrogates TRAIL resistance in leukemia cells Blood 2006 107 679 688 10.1182/blood-2005-05-1982 16195335

[B10] Su WC Chang SL Chen TY Chen JS Tsao CJ Comparison in in vitro growth-inhibitory activity of carboplatin and cisplatin on leukemic cells and hematopoietic progenitors: the myelosuppressive activity of carboplatin may be greater than its antileukemic effect Jpn J Clin Oncol 2002 30 562 567 10.1093/jjco/hyd137 11210167

[B11] Jung EM Lim JH Lee TJ Park JW Choi KS Kwon TK Curcumin sensitizes tumor necrosis factor-related apoptosis-inducing ligand (TRAIL)-induced apoptosis through reactive oxygen species-mediated upregulation of death receptor 5 (DR5) Carcinogenesis 2005 26 1905 1913 10.1093/carcin/bgi167 15987718

[B12] Yodkeeree S Sung B Limtrakul P Aggarwal BB Zerumbone enhances TRAIL-induced apoptosis through the induction of death receptors in human colon cancer cells: Evidence for an essential role of reactive oxygen species Cancer Res 2009 69 6581 6589 10.1158/0008-5472.CAN-09-1161 19654295 PMC2741416

[B13] Mahalingam D Szegezdi E Keane M de Jong S Samali A TRAIL receptor signalling and modulation: Are we on the right TRAIL? Cancer Treat Rev 2009 35 280 288 10.1016/j.ctrv.2008.11.006 19117685

[B14] Kelley SK Ashkenazi A Targeting death receptors in cancer with Apo2L/TRAIL Curr Opin Pharmacol 2004 4 333 339 10.1016/j.coph.2004.02.006 15251125

[B15] Rosato RR Almenara JA Dai Y Grant S Simultaneous activation of the intrinsic and extrinsic pathways by histone deacetylase (HDAC) inhibitors and tumor necrosis factor-related apoptosis-inducing ligand (TRAIL) synergistically induces mitochondrial damage and apoptosis in human leukemia cells Mol Cancer Ther 2003 2 1273 1284 14707268

[B16] Martin SJ Reutelingsperger CP McGahon AJ Rader JA van Schie RC LaFace DM Green DR Early redistribution of plasma membrane phosphatidylserine is a general feature of apoptosis regardless of the initiating stimulus: inhibition by overexpression of Bcl-2 and Abl J Exp Med 1995 182 1545 1556 10.1084/jem.182.5.1545 7595224 PMC2192182

[B17] Rezácová M Vávrová J Vokurková D Ionizing radiation sensitizes leukemic MOLT-4 cells to TRAIL-induced apoptosis Acta Medica 2008 51 101 105 18998361 10.14712/18059694.2017.10

[B18] Gélinas C White E BH3-only proteins in control: specificity regulates MCL-1 and BAK-mediated apoptosis Genes Dev 2005 19 1263 1268 10.1101/gad.1326205 15937216

[B19] Clohessy JG Zhuang J de Boer J Gil-Gómez G Brady HJ Mcl-1 interacts with truncated Bid and inhibits its induction of cytochrome c release and its role in receptor-mediated apoptosis J Biol Chem 2006 281 5750 5759 16380381 10.1074/jbc.M505688200

[B20] Galluzzi L Vitale I Abrams JM Alnemri ES Baehrecke EH Blagosklonny MV Molecular definitions of cell death subroutines: recommendations of the nomenclature committee on cell death 2012 Cell Death Differ 2012 19 107 120 10.1038/cdd.2011.96 21760595 PMC3252826

[B21] Budd RC Yeh WC Tschopp J cFLIP regulation of lymphocyte activation and development Nat Rev Immunol 2006 6 196 204 10.1038/nri1787 16498450

